# Targeted Interventions in HAM/TSP: Emerging Therapies and Future Directions – A Narrative Review

**DOI:** 10.1002/hsr2.72863

**Published:** 2026-07-30

**Authors:** Meygol Mirzaei Rezaei, Mahdi Khosravi Nia, Kasra Allaei Rouzbahani, Maryam Kazemi, Negar Asghari Hosori, Mehdi Norouzi, Farzaneh Sotoudegan, Narges Eslami, Negar Ariamand, Vahid Shahnavaz, Arash Letafati, Sayed‑Hamidreza Mozhgani

**Affiliations:** ^1^ Research Center for Clinical Virology Tehran University of Medical Sciences Tehran Iran; ^2^ Department of Medical Genetics, Faculty of Medicine Hormozgan University of Medical Sciences Bandar Abbas Iran; ^3^ Cellular and Molecular Research Center, Basic Health Sciences Institute Shahrekord University of Medical Sciences Shahrekord Iran; ^4^ Department of Virology, School of Public Health Tehran University of Medical Sciences Tehran Iran; ^5^ Quality Control of Medicines and Supplements Group, Pharmaceutical Quality Assurance Research Center The Institute of Pharmaceutical Sciences (TIPS) Tehran Iran; ^6^ Student Research Committee Alborz University of Medical Sciences Alborz Iran; ^7^ Department of Microbiology and Virology, School of Medicine Alborz University of Medical Sciences Alborz Iran; ^8^ Non‐Communicable Disease Research Center Alborz University of Medical Sciences karaj Iran

**Keywords:** HAM/TSP, HTLV‐1, retroviruses, treatment

## Abstract

**Background and Aim:**

Human T‐Lymphotropic virus type 1 (HTLV‐1) infection can lead to HTLV‐1‐associated myelopathy/tropical spastic paraparesis (HAM/TSP), a debilitating chronic neurological disease characterized by progressive lower limb spasticity and autonomic dysfunction. Because the exact disease pathogenesis remains unclear and current management options have limited efficacy, there is a critical need for more effective therapies to reduce morbidity and mortality. This narrative review aims to review the available and diverse range of emerging treatment approaches for HAM/TSP.

**Methods:**

A comprehensive narrative review of the existing literature was conducted to assess current treatment methods, historical treatment efforts, and modern therapeutic interventions. The scope includes pharmacological, immunological, targeted, and non‐pharmacological strategies aimed at disease management.

**Results:**

Current disease‐modifying therapies primarily rely on immunomodulatory agents, such as corticosteroids and interferon‐alpha, to mitigate neuroinflammation and preserve motor function, whereas traditional antiretroviral therapies demonstrate limited clinical efficacy. Emerging targeted therapies, notably the monoclonal antibody mogamulizumab, show significant promise in reducing HTLV‐1‐infected cells and sustaining a decrease in spinal cord inflammation. Furthermore, comprehensive care must integrate treatments targeting specific cellular signaling pathways alongside vital non‐pharmacological interventions, such as physical rehabilitation and psychological support.

**Conclusion:**

The effective management of HAM/TSP necessitates a holistic, multidisciplinary approach encompassing targeted pharmacotherapy, symptomatic management, and psychosocial support. While novel interventions and disease‐modifying therapies offer promising new directions, further high‐quality, randomized clinical trials are essential to establish standard regimens, optimize dosages, and ensure long‐term efficacy and safety.

## 1 Introduction to HAM/TSP, Historical Overview: Past Approaches to HAM/TSP Treatment

HTLV‐1‐Associated Myelopathy/Tropical Spastic Paraparesis (HAM/TSP) was initially identified in the 1980s, primarily affecting individuals infected with Human T‐lymphotropic virus 1 (HTLV‐1) [1]. HTLV‐1 infects an estimated 5–20 million people worldwide, with endemic prevalence in Japan (0.1–1% seroprevalence, but with a much higher incidence in the Kyushu region), Central and Northern Australia (up to 40% in Indigenous populations), and the Caribbean and South America [2, 3]. The prevalence of antibodies targeting this virus is relatively low in North America and Europe, with reported rates of 0.01–0.03% in Canada and the United States, 0.0056% in Greece, and 0.002% in Norway [4].

HAM/TSP typically manifests as progressive spastic paraparesis characterized by weakness, muscle stiffness, and difficulty walking. Additionally, patients may experience bladder dysfunction, sensory disturbances, and lower limb muscle atrophy. The pathogenesis is multifactorial and involves factors related to both the virus and host [5, 6]. In the initial stages of HAM/TSP research, diagnostic challenges posed significant obstacles in accurately identifying and differentiating HAM/TSP from other neurological conditions [7, 8].

A pioneering study by Osame et al. in 1986 played an essential role in characterizing HAM/TSP as a distinct clinical entity. The authors described the clinical features and proposed diagnostic criteria, emphasizing the importance of a thorough medical history, neurological examination, and laboratory investigation [9, 10].

Serological methods, including Western blotting and enzyme‐linked immunosorbent assays (ELISAs) for HTLV‐1 antibody detection, were applied to confirm HAM/TSP‐suspected patients [11]. Despite serological validation of anti‐HTLV‐I antibodies, further investigations are required to differentiate HAM/TSP from other neurological disorders with similar clinical presentations. Cerebrospinal fluid (CSF) analysis became an important diagnostic sample, revealing lymphocytic pleocytosis, elevated protein levels, and oligoclonal bands in HAM/TSP cases [1, 12, 13, 14].

Polymerase chain reaction provides an opportunity to directly identify viral DNA in peripheral blood mononuclear cells (PBMCs) or CSF samples [15, 16, 17].

Early treatment efforts focused on improving patients' neurological function with immunomodulatory treatments, such as steroid therapy, physical therapy, and assistive devices, which were commonly employed to maintain or improve functional capabilities [18, 19].

In addition to symptomatic management, HIV‐antiretroviral therapy (ART), such as zidovudine (AZT), can be effective to some extent in HAM/TSP patients; however, the effectiveness of ART remains controversial, as not all patients respond equally to antiretroviral drugs [20]. Factors such as disease duration, viral proviral load, and immune response variations may influence therapeutic outcomes [21].

## 2 Antiretroviral Therapy

Antiretroviral therapy (ART) has revolutionized the management of HIV infection, significantly improving the prognosis and quality of life of HIV‐positive individuals. Consequently, researchers have explored the potential of HIV antiretroviral drugs in treating HTLV‐1‐associated HAM/TSP [21].

Several studies have investigated the implementation of AZT, a nucleoside reverse transcriptase inhibitor (NRTI), in patients with HAM/TSP [22, 23]. Zidovudine suppresses HTLV‐1 Gag protein production and may reduce proviral DNA in HTLV‐1 exposed‐primary CD4 + T lymphocytes [24]. Low concentrations of zidovudine (0.1 μM) inhibit HTLV‐1 transmission to adult PBMCs in vitro and interrupt HTLV‐1 DNA and RNA production [25]. Lamivudine (3TC) and Zidovudine (ZDV) belong to the class of NRTIs, which work by inhibiting the enzyme reverse transcriptase, which is vital for HIV replication, thereby reducing the virus load in the body [26]. Although some improvements have been found in the aforementioned results of the antiretroviral drugs, zidovudine–lamivudine co‐therapy mainly could not improve the clinical symptoms (Table 1). Lamivudine is classified as a synthetic nucleoside analog. Previously referred to as 3TC, lamivudine is used in conjunction with zidovudine to manage HIV infection in individuals facing disease progression [32]. Therefore, the HAM‐TSP Practical Guidelines are not recommended the administration of antiretroviral drugs [21]. Raltegravir, an integrase inhibitor antiretroviral, prevents HTLV‐1 cell‐to‐cell transmission in vitro. Raltegravir did not decrease proviral load in the patient cohort; however, in a subset of patients, it led to a reduction in proviral load, Tax and HBZ mRNA expression, along with a decrease in CD4 + CD25 + T cells, and spontaneous lymphoproliferation was observed [33].

**Table 1:** Summary of studies on HTLV‐1 zidovudine–lamivudine co‐therapy.

Year and Ref	Dosage (AZT, lamivudine)	Result
1999 [27]	2000,150 mg	A 10‐fold decrease was observed in the HTLV‐1 proviral load.
2001 [28]	NA/NA	One patient experienced a 2‐log reduction in HTLV‐I proviral load, whereas another patient showed a 1‐log increase.
2002 [29]	6.25 µM lamivudine	Although 3TC does not strongly suppress HTLV‐1 replication, its ability to inhibit long‐term cell growth may still have therapeutic relevance by limiting disease progression or transformation into leukemia/lymphoma.
2006 [30]	300, 150 mg	10‐fold reduction in the proviral load but did not result in significant clinical improvements.
2013 [31]	300/150 mg	Patient's lower extremity weakness had much improved.

Despite some observed effects against HTLV‐1, none of the studies performed in HAM‐TSP patients associated ART administration with an enhanced clinical score, and the 2019 guidelines by Araujo et al. did not advise the use of antiretroviral drugs for managing HAM‐TSP [21].

## 3 Immunomodulatory Therapies

The main therapeutic option for HAM/TSP treatment is immunomodulatory agents, such as corticosteroid hormones and interferon‐α [34, 35]. IFN‐α, an immunomodulatory cytokine, has also been explored as a potential treatment [36]. It may modulate immune dysregulation and suppress viral replication, thereby mitigating neurological damage [37]. In a clinical trial, IFN improved motor dysfunction in almost 70% of HAM/TSP patients, and even urinary effectivity persisted for 4 weeks post‐treatment disturbances in some cases [38]. Subsequent studies have further supported the potential benefits of IFN‐α, indicating a decrease in proviral load and progression of neurological manifestations [36]. One study found that leukocyte‐derived IFN‐α/β was effective in restricting enteric viral infections in mice [39]. While in a study in 1996, IFN‐alpha and ascorbic acid (AA) at high doses had a modest clinical efficacy in HAM/TSP patients, in another study by Britta et al., AA had a higher anti‐proliferative, antiviral, and immunomodulatory activity than IFN‐alpha in HTLV‐1 infected cell lines and HAM/TSP PBMC [40, 41]. Due to its disease‐modifying effect, interferon‐alpha is still considered a therapeutic option for HAM/TSP patients, mainly because of the immunopathological nature of the disease [42]. Despite some evidence of its efficacy in patients, clinical approval of this treatment has only been issued in Japan since 2000, and there is no FDA approval for managing HAM/TSP with IFN‐α [35, 43]. Corticosteroid treatment is the most widely used therapeutic option for patients with HAM/TSP. Corticosteroids are potent anti‐inflammatory agents that suppress immune responses [44]. Corticosteroids are administered to sustain motor function by suppressing inflammation [45, 46]. Corticosteroids bind to glucocorticoid receptors in immune cells, resulting in the inhibition of inflammatory responses by modulating cytokines and chemokines [47]. However, prolonged corticosteroid therapy may be associated with serious side effects, such as osteoporosis, adrenal insufficiency, gastrointestinal, hepatic, aseptic joint necrosis, ophthalmologic effects, hyperlipidemia, growth suppression, and possible congenital malformations [48]. Observational studies have reported diverse results regarding the use of corticosteroids in HAM/TSP. Some studies have found beneficial effects on clinical outcomes, such as motor function, spasticity, pain, bladder control, and viral load [21, 49]. Currently, the administration of high‐dose methylprednisolone and oral prednisolone (5 mg) is considered the most effective treatment option for managing HAM/TSP, according to international consensus guidelines and recommendations [50]. According to a multicenter retrospective cohort study, daily PSL treatment is effective in slowing the progression of HAM/TSP [51].

Azathioprine is an immunosuppressive drug that has been used to manage HAM/TSP patients. Treatment of nine patients with azathioprine at 50–100 mg daily for 1–3 months improved motor disability to some extent [41].

## 4 Monoclonal Antibodies

Monoclonal antibodies have also been used to manage patients with HAM/TSP. Monoclonal antibodies are laboratory‐produced molecules that can target specific cells or proteins in the immune system [21]. Interleukin‐2 (IL‐2) is a cytokine that stimulates the proliferation of T cells and natural killer cells [52]. Hu‐Mikβ1, a humanized anti‐IL‐2/IL‐15 receptor β chain, inhibited aberrant CD8 + T cell proliferation and IFN‐γ expression, particularly in HAM/TSP patients with CD122 saturation. However, no clinical efficacy was observed [1].

### 4.1 ANTI CD3

Anti‐CD3 monoclonal antibodies have been proposed as a possible therapeutic option in HAM/TSP treatment. Anti‐CD3 mAb showed immunomodulatory effect through induction of apoptosis, reduction of aberrant lymphoproliferation, and anti‐inflammatory gene expression stimulation, and/or immunoregulatory cell proliferation [53].

HAM/TSP patients have a major infiltration of HTLV‐1‐infected CCR4+ cells to their spinal cord, which, through IFN‐γ production, stimulates the secretion of CXCL10 from astrocytes, leading to the recruitment of CXCR3+ cells, such as inflammatory cells, to the spinal cord and chronic inflammation persistence [54].

A study has revealed that HTLV‐1 infection leads to chromatin remodeling, which affects CD3 genes. Chromatin remodeling refers to the structural changes in chromatin that can regulate gene expression. In this case, it results in the progressive loss of CD3 expression on the surface of the infected T‐cells. The loss of CD3 expression begins with a decrease in CD3 γ mRNA, followed by reductions in CD3 δ, CD3 ε, and CD3 ζ mRNA. This sequential reduction suggests stepwise silencing of the CD3 genes. This study suggests that the loss of CD3 expression is caused by an epigenetic mechanism. Epigenetic changes are alterations that affect gene expression without changing the DNA sequence. In this case, the chromatin around the CD3 genes becomes less accessible to the transcription machinery, leading to reduced gene expression. Interestingly, the loss of CD3 expression continued even after silencing of early viral genes. This indicates that epigenetic changes are stable and persist over time. This study raises the possibility that the progressive loss of CD3 expression could be linked to the eventual malignant transformation of T‐cells. However, further research is required to establish whether this loss directly contributes to the development of adult T‐cell leukemia/lymphoma (ATLL) or is merely an associated phenomenon [55].

### 4.2 MOGAMULIZUMAB

Mogamulizumab functions by specifically targeting and reducing CCR4 + T cells, which are primarily infected with HTLV‐1. The decrease in the number of infected cells is intended to reduce chronic inflammation and immune‐related damage in the spinal cord. An extended study following the initial phase 1–2a trial was conducted to assess the long‐term safety and effectiveness of the anti‐CCR4 antibody mogamulizumab in patients with HAM/TSP. The results indicated that prolonged treatment with mogamulizumab was well tolerated. This treatment effectively reduced the number of HTLV‐1‐infected cells in both peripheral blood and cerebrospinal fluid (CSF) and maintained a decrease in spinal cord inflammation for more than 4 years. Clinical results have demonstrated that mogamulizumab consistently improves spasticity and urinary dysfunction, particularly alleviating obstructive symptoms in patients with HAM/TSP. Additionally, when compared to a contemporary control group, the treatment potentially slowed the progression of motor impairment in the lower limbs. Long‐term administration of mogamulizumab was found to be generally safe and well‐tolerated in patients with HAM/TSP. This finding was supported by the observation that 15 out of 21 participants (71%) successfully received multiple doses of the medication over 4 years. Although 17 out of 21 participants (81%) experienced a high incidence of mild to moderate skin‐related adverse events, such as skin rash and herpes zoster, more severe skin reactions, like Stevens‐Johnson syndrome and toxic epidermal necrolysis, known to occur in ATLL, were not observed. Additionally, neutralizing antibodies to mogamulizumab were found in 4 of the 21 participants (approximately 19%), but no cases of anaphylactic shock or infusion reactions were reported with premedication during repeated administration [56].

Mogamulizumab is a humanized antibody with cytotoxic effects on CCR4+ cells and is used to treat ATLL. A clinical trial demonstrated that mogamulizumab treatment reduced the HTLV‐1 proviral load in vivo and alleviated neurological symptoms in patients with HAM/TSP [57]. Recent research by Sato et al. suggests that mogamulizumab may slow the progression of lower limb motor disability in HAM/TSP patients who were also treated with corticosteroids [56].

Bladder and bowel problems, lower limb spasticity, weakness, sexual dysfunction, and back pain are the principal symptoms of HAM/TSP, which can affect the patients' physical, mental, and social quality of life [58]. The two main strategies for treating HAM/TSP are disease‐modifying therapies (DMT) and symptomatic therapies [59]. DMT aims to reduce viral load and immune‐mediated damage in the spinal cord using antiviral drugs, immunosuppressants, or immunomodulatory agents [21, 45]. However, evidence for the effectiveness and safety of DMT is scarce and conflicting, and there is no agreement on the optimal treatment regimen or its duration [21].

## 5 Alemtuzumab

Alemtuzumab is a monoclonal antibody that targets CD52, a protein found in T cells. It has been utilized in the treatment of HAM/TSP and has demonstrated effectiveness in lowering the population of T cells infected with HTLV‐1 and improving clinical symptoms. However, Alemtuzumab can cause serious side effects, including infections and autoimmune disorders [60, 61].

## 6 Modulating Immune Responses

IL‐2 is a cytokine that promotes the proliferation and activation of T cells and natural killer cells. IL‐2 has been used as an immunomodulatory agent in patients with low proviral load and mild symptoms. A study showed that IL‐2 treatment led to a rise in the frequency of regulatory T cells and a decrease in the frequency of activated T cells in patients. The study also reported an enhancement in motor function and overall quality of life in certain patients. However, prolonged usage in a chronic illness, like HAM/TSP may lead to severe complications [62].

IL‐10 is a cytokine that acts as an anti‐inflammatory agent, and inhibits inflammatory reactions by suppressing pro‐inflammatory cytokine production. IL‐10 has been investigated as an immunomodulatory agent with high proviral load and severe symptoms. Its treatment results in a decrease in the levels of pro‐inflammatory cytokines in cerebrospinal fluid and motor function improvement and bladder control in some patients. However, IL‐10 treatment was associated with a side effect of thrombocytopenia [63, 64]. IL‐17 is a cytokine that promotes inflammatory responses and tissue damage in HAM/TSP. IL‐17 inhibitors block the binding or signaling of IL‐17 or its receptor and are a possible treatment target. Nevertheless, additional research is required to assess the effectiveness and safety [65]. TNF‐alpha, is a pro‐inflammatory cytokine that mediates inflammatory responses and tissue damage in HAM/TSP. TNF‐alpha inhibitors block the binding or signaling of TNF‐alpha or its receptor and are a potential therapeutic target for HAM/TSP. Additional investigation is required to determine its efficacy and safety [66].

Vaccines for HTLV‐1 have been proposed as a preventive or therapeutic strategy for HTLV‐1 infection and related conditions. Vaccine candidates are based on viral proteins or peptides and aim to induce HTLV‐1‐specific antibodies or cytotoxic T cells. Some vaccine candidates have shown efficacy in animal models or phase I clinical trials. However, further studies are needed to determine their safety and efficacy [67, 68].

The selection and dosage of these drugs should be customized to each patient's condition and response, and possible adverse effects should be checked regularly [21, 69]. Non‐pharmacological interventions include interventions that aim to improve the physical, functional, emotional, and social well‐being of patients with HAM/TSP. These interventions include physical therapy, occupational therapy, psychological counseling, and other techniques [70, 71].

## 7 Herbal Treatments

### 7.1 Curcumin

Curcumin is a bioactive compound derived from the Curcuma longa L (turmeric) plant that has demonstrated antiviral effects on retroviruses, especially HTLV‐1, and does not exhibit toxic effects on the viability of human cell lines. Retroviruses contain three key enzymes: protease (PR), reverse transcriptase (RT), and integrase (IN). HTLV‐1 protease, a homodimeric protein, plays a crucial role in cleaving HTLV‐1 polyproteins during infection. As an HTLV‐1 protease inhibitor, curcumin can bind to the active site of HTLV‐1 protease, interacting with multiple residues within this region. However, the curcumin compound is not considered a suitable candidate for use as a therapeutic approach because of its poor solubility and bioavailability. However, curcumin metabolites, such as curcumin‐glucuronide, tetrahydro‐curcumin, and curcumin‐sulfate present promising alternatives with desirable performance [72].

### 7.2 Bidens Pilosa

Bidens pilosa, a tropical plant, shows a high potential for the treatment of HTLV‐1, especially adult T‐cell leukemia/ lymphoma (ATLL). The extract of this plant inhibits the growth of HTLV‐1‐infected T‐cell lines by inducing apoptosis. It can cause cell cycle arrest in the G1 phase and prevent progression to the S phase. In addition, it affects the proliferation of ATLL cells by inhibiting the activation of NF‐κB by reactive oxygen species (ROS) and reducing the phosphorylation of IκB kinase β and IκBα. B. pilosa extract suppresses the expression of AP‐1 components like JunB and JunD, further inhibiting cell growth [73]. More studies most be done in the context of HAM/TSP to see if it has similar effect to this disease or not.

### 7.3 Green Tea Polyphenols

Green tea polyphenols, especially epigallocatechin‐3‐gallate (EGCg), exhibit significant cytotoxic inhibitory effects against HTLV‐1 by inducing apoptosis pathways and downregulating key viral oncogenes. The growth of peripheral blood lymphocytes of ATL is significantly inhibited by inducing apoptosis in HTLV‐1‐infected cells by concentrations of green tea extract (TEA) and EGCg in the range of 3–27 μg/mL, whereas normal PBLs show negligible apoptosis when exposed to the same concentrations of TEA or EGCg. Furthermore, in ATL PBLs, the expression of the HTLV‐I pX gene, which is critical for the oncogenic activity of the virus, is suppressed by more than 90% following the application of TEA and EGCg. This suppression occurs without a significant effect on β‐actin gene mRNA expression, indicating a targeted action against viral gene expression while preserving normal cellular function [74].

### 7.4 Eucalyptus Camaldulensis

Eucalyptus camaldulensis has shown potential in the treatment of human T‐cell leukemia virus type 1 (HTLV‐1) due to its active compounds. Research shows that the ethanolic extract of its leaves inhibits the activities of the viral oncoprotein Tax, which is critical for HTLV‐1 pathogenesis and plays a major role in the development of ATL. The extract specifically reduces the activation of Tax‐induced transcription factors such as NF‐kB and serum response factor in Jurkat cells, a human T‐cell line. This mechanism involves preventing the degradation of IκBα, a protein that retains NF‐kB in the cytoplasm, thereby blocking its translocation to the nucleus. While Eucalyptus camaldulensis does not directly affect the binding of Tax to NF‐κB, it disrupts its interaction with IKKγ, which is essential for its activity. Notably, the 40% methanolic fraction of the extract, which is rich in polyphenols, exhibited the most potent inhibitory effects on Tax activities [75].

### 7.5 Scutellaria baicalensis Georgi

Scutellaria baicalensis Georgi, a traditional Chinese medicinal plant, contains the flavonoid baicalin, which has shown significant potential in the treatment of HTLV‐1. Research shows that baicalin can inhibit HTLV‐1 replication in infected T and B cells and achieve a greater than 70% reduction in p19 gag protein levels, without adversely affecting cell viability or total cellular protein synthesis. Baicalin effectively reduces the activity of reverse transcriptase (RT), an enzyme critical for retroviral replication, thereby inhibiting the viral replication mechanism. Furthermore, peripheral blood lymphocytes that were pre‐treated with baicalin exhibit resistance to HTLV‐1 infection when exposed to infected cells [76].

### 7.6 Momordica Charantia

Momordica charantia exhibits significant inhibitory effects on HTLV‐1 through its various plant compounds. In vitro studies showed that both alcoholic and aqueous extracts of M. charantia significantly reduced HTLV‐1 proviral load (PVL) in human umbilical vein endothelial cells at concentrations of 5, 10, and 20 μg/mL while increasing the sensitivity of immune system in mesenteric lymph nodes to HTLV‐1 infection. Furthermore, the rich compounds of M. charantia make it a valuable candidate for the development of new therapeutic approaches for HTLV‐1‐associated diseases, including ATLL and HAM/TSP [77].

## 8 Other Pharmacological Interventions

These are some less pharmacological interventions that have been studied to treat HAM/TSP. Prosultiamine, a derivative of vitamin B1, improved neurological symptoms, including motor dysfunction of the lower extremities and urinary disturbance, and reduced HTLV‐1 proviral load [34]. Pentoxifylline (PTX), an anti‐inflammatory drug significantly suppressed TNF‐α spontaneous production in HAM patients PBMCs and trivial inhibited GM‐CSF and IFN‐γ production [78].

The findings of research indicate that danazol has a beneficial impact on alleviating motor disabilities and symptoms in patients with HAM/TSP, particularly when evaluated against its comparatively lower side effects about alternative treatments like corticosteroids, as well as its cost‐effectiveness for specific patient populations. As an immunomodulatory agent, danazol demonstrates a notable capacity to enhance motor function and mitigate symptoms in individuals affected by HAM/TSP [79].

A study suggests that cyclosporine could be beneficial for patients with recently developed HAM/TSP or those with chronic HAM/TSP who are experiencing a recurrence. Nonetheless, the long‐term effects and safety profile of this steroid‐sparing treatment still need to be clarified and warrant further research. Notably, cyclosporine A, an immunosuppressive agent, not only enhanced the clinical status of the patient with HAM/TSP but also contributed to a reduction in HTLV‐1 proviral load, all while exhibiting minimal toxicity [80].

It is crucial to highlight that the efficacy and safety of these pharmacological approaches may vary, and further research is needed to establish their role in the management of HAM/TSP. Table 2 outlines key pharmacologic treatments and highlights clinical efforts aimed at addressing HTLV‐1 infection.

**Table 2:** Therapeutic strategies and clinical trials in HTLV‐1 management.

Sample Size	Test Type	Drug(s)	Result	Year(s)	Ref
66	Phase 3 RCT double‐blind + open‐label extension	Mogamulizumab	Secondary: ↓HTLV‐1 PVL (~60%, *p* < 0.001) ↓CSF neopterin/CXCL10.	2024 (completed, not terminated)	[81]
21	Phase 1/2a open‐label + extension	Mogamulizumab	↓PVL 65% (*p* < 0.001) spasticity improvement 95% OMDS improvement 22%	2018	[82]
16 (ex vivo)	Phase I/II pilot	Teriflunomide	↓HTLV‐1‐infected CD4 + T‐cell proliferation (IC50 = 14 μM) Suppressed Tax expression modulated IL‐2/STAT5	2021	[83]
20	Phase 2 open‐label study	l‐ARGININE	↑ walking speed ↓ TUGT time ↓ CSF neopterin	2022	[84]
24	Open‐label pilot study	Prosultiamine (Vitamin B1 derivative)	Improved bladder symptoms 12 weeks	2013	[85]
13	Pilot clinical trial (Iran)	VALPROATE + PEG‐IFN + PREDNISOLONE	↓ HTLV‐1 PVL & Tax/HBZ mRNA Improved clinical outcomes regimen well‐tolerated	2015	[86]

## 9 Stem Cell Transplantation

Stem cell transplantation offers a potential treatment option for individuals affected by HAM/TSP. Allogeneic hematopoietic stem cell transplantation (allo‐HSCT) has demonstrated curative potential for ATLL; however, the presence of comorbidities can elevate the risk of mortality associated with the transplant procedure. Administration of allo‐HSCT in a 48‐year‐old man with ATLL with HAM/TSP with a history of prednisolone therapy caused Mild gait improvement (OMDS 5) on Day 30. Interestingly HAM/TSP symptoms progression was not clear after donor lymphocyte infusions which suggested that ATLL patients with HAM/TSP might tolerate allo‐HSCT and donor lymphocyte infusions [87].

Another study conducted across seven institutions in Japan from 1997 to 2002 examined 40 cases of allo‐HSCT for ATLL. All patients who were evaluable achieved complete remission (CR) following the allo‐HSCT procedure, with a median survival duration of 9.6 months for the entire cohort. Among the ten patients who experienced a relapse of ATLL, five were able to attain CR once more; notably, three of these cases responded positively to the reduction or discontinuation of immunosuppressive therapy. These findings imply that allo‐HSCT may be a viable treatment option for certain patients with aggressive forms of ATLL [88]. In addition to that, another retrospective analysis involving 35 patients who faced either progression or relapse of persistent ATLL following their initial allogeneic stem cell transplantation (allo‐SCT) was conducted across three medical institutions in Nagasaki prefecture, Japan, during the period from 1997 to 2010. Among these patients, 29 underwent a treatment strategy that involved the cessation of immunosuppressive therapy as the primary intervention, leading to CR in two individuals. The findings of this study indicate that the activation of a graft‐versus‐ATL response may be essential for achieving long‐lasting remission in ATLL patients who experience relapse or disease progression after allo‐SCT [89]. Although these findings are encouraging, they primarily reflect outcomes in ATLL rather than HAM/TSP.

## 10 Physical Therapy

Physical therapy exerts certain types of exercises, massages, and treatments based on physical stimuli such as heat, cold, electrical currents, or ultrasound for relieving pain, and strengthen weakened muscles [90].

Due to the considerable motor disabilities, patients with neurological complications associated with HAM/TSP are recommended to undergo physical therapy, which enhances their functional abilities, reduces symptoms, and positively influences their quality of life [91, 92]. It has been shown that Pilates might be an effective auxiliary physical treatment for HAM/TSP patients. Pilates significantly enhanced pain levels, static and dynamic balance, trunk control, and mobility, and simultaneously and notably reduced of the cytokines IFN‐γ and IL‐10 serum levels. Interestingly, 10 weeks after discontinuing Pilates, pain and mobility worsened significantly [93]. Another study demonstrated that following the physical therapy guidelines of the International Classification of Functioning, Disability, and Health (ICF) improved quality of life and reduced disease morbidity. The neuromuscular facilitation technique significantly reduced abnormal sensory input and motor neuron activity, with a more substantial effect when proprioceptive input was involved [94].

## 11 Psychological Support

Psychiatric disorders contribute to chronic disease patients' comorbidity, such as individuals suffering from HAM/TSP [95]. Depressive symptoms might also contribute to the progression of functional disabilities and decrease patients' quality of life [96].

Viral infection might contribute to neuronal system dysfunction initiation, causing behavioral abnormalities. Recent studies have supported the role of viral infection in the development of psychiatric disorders [97, 98].

Previous studies showed a relationship between HTLV‐1 infection and depression and anxiety. It has been shown that HAM/TSP patients are more prone to develop depression and anxiety than HTLV‐1 carriers [96, 99]. Their depression could be linked to psychological effects of the viral infection or the direct biological impact of the virus [100].

In a case report by Sohler et al., the severe progression of HAM/TSP resulting in paraplegia was found to contribute to intense depression. This mental disturbance subsequently led to psychogenic movement disorder [100].

To sum up, Psychiatric conditions, including depression and anxiety, are commonly observed among patients with HAM/TSP, exacerbating their disabilities and diminishing their overall quality of life. It is essential for future initiatives to prioritize early detection, implement integrated care that encompasses both mental and physical health aspects, investigate the underlying biological mechanisms, and establish robust support systems aimed at improving patient outcomes and enhancing overall well‐being.

## 12 Current Challenges and Future Directions

Currently, there are no licensed vaccines or antiviral agents against HTLV‐1 infection, and a promising treatment with significant improvement of neurological functions has not been introduced. Due to the unsatisfactory results of therapeutic option against the virus, the main strategy of clinical management is symptomatic treatment to relieve pain, spasticity, and urinary symptoms [45, 101].

Because of the neuroinflammatory nature of the disease immunomodulatory agents such as IFN‐α have been used to mitigate the disease symptoms. INF‐α is a tested treatment option of HAM/TSP that improved neurological symptoms [38]. However, based on the Japanese national registry of HAM/TSP patients, IFN‐α is currently used by only a small percentage of patients, likely due to the limited evidence supporting its long‐term benefits and the potential for various adverse effects [5].

Corticosteroids are the most widely recommended treatment for HAM/TSP, despite the absence of randomized, controlled trials. Several observational studies have shown that corticosteroids can improve motor function [41, 49]; but, other studies suggest that the treatment's effectiveness is limited, with some patients continuing to experience motor dysfunction progressing despite therapy [35]. The use of corticosteroids tends to be more beneficial for patients with shorter disease duration and higher inflammatory activity [102]. A recent retrospective study, spanning a 3.4‐year follow‐up period, compared 57 patients on continuous low‐dose corticosteroid therapy with 29 untreated individuals. The findings revealed that motor function deterioration occurred at a slower rate in the group receiving steroid treatment [5, 51]. A randomized, placebo‐controlled multicenter trial investigating oral prednisolone therapy is currently being conducted in Japan (UMIN000024086). Additionally, cyclosporine A, a calcineurin inhibitor, was evaluated in a pilot study with seven participants, showing signs of clinical improvement and suggesting its potential as an alternative to steroids [5]. This can also be further studied for HTLV‐1 infection too.

To address the challenges of treatment, several future directions have been proposed, including: Conduct high‐quality clinical trials on DMTs for HAM/TSP: To generate robust and reliable evidence on the efficacy and safety of DMTs for HAM/TSP, high‐quality clinical trials are needed. Moreover, the outcome measures used in these trials should be standardized and validated and should encompass various aspects of HAM/TSP, such as disability, quality of life, biomarkers, and imaging [35]. Comparative studies should also be conducted to identify the most effective and safe treatment options for HAM/TSP.

## 13 Novel Therapeutic Strategies on the Horizon

### 13.1 Targeting Cellular Pathways

Several approaches have been explored to achieve this goal, such as using monoclonal antibodies that bind to specific surface molecules on infected cells (e.g., CCR4, CD25, CD52), using small molecule inhibitors that block key signaling pathways in HTLV‐1‐infected cells (e.g., JAK3, NF‐kB, PI3K/AKT), or using gene therapy that introduces suicide genes or anti‐HTLV‐1 genes into infected cells [103, 104, 105, 106, 107]. Some of these strategies have demonstrated encouraging outcomes in preliminary investigations or medical experiments. However, additional inquiry is required to enhance their effectiveness, security, and administration.

The signaling network map for ATLL and HAM/TSP visually compares the important genes found in each disease. In ATLL, there's an upregulation in the expression of genes including NGF, NFAT, PRKCB, CLAM1, PI3K‐AKT, AP1, and TNF, which is alongside by the spontaneous activation of the NF‐κB pathway and persistent lymphocyte activation. Genes like PRKCB, ITGA2, IL8, and NOS2 show increased expression, playing a role in processes such as inflammation, cell survival, angiogenesis, and migration. In contrast, HAM/TSP pathways linked to apoptosis and immune dysregulation are activated. Genes like TP53, EGR1, Serpine1, and IGFR1 promote apoptosis, while the upregulation of MAP3K1, TLR2/4, STAT3, CREB1, and APP contributes to immune response disruption [108].

### 13.2 NF‐κB Pathway

**Figure 1:**
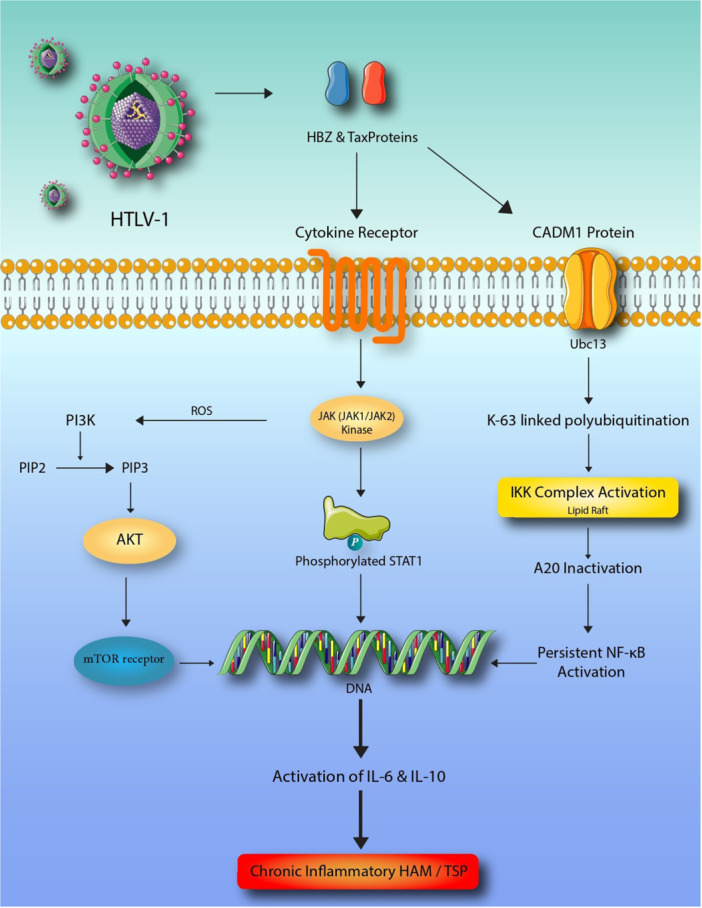
The figure illustrates the HTLV‐1 induced inflammatory conditions via NF‐KB, JAK‐STAT, and PI3K‐AKT signaling pathways. It depicts how HTLV‐1 interacts with cytokine receptors and the CADM1 protein, initiating a cascade of molecular events. HBZ and Tax are crucial viral proteins in the HTLV‐1 virus life cycle and its associated pathologies. The pathway includes the conversion of PIP2 to PIP3 by PI3K, which activates AKT, leading to the stimulation of the mTOR receptor. Additionally, JAK kinase phosphorylates STAT, resulting in DNA activation and the production of IL‐6 and IL‐10, both of which play roles in chronic inflammatory HAM/TSP. The image also emphasizes the activation of the IKK complex, inactivation of A20, and persistent activation of NF‐KB in this process. In HAM/TSP, the Tax protein from HTLV‐ 1 activates this pathway resulting in higher levels of pro‐inflammatory cytokines such as TNF‐a, IL‐1ẞ, and IL‐6. This results in persistent inflammation, which damages neurons and glial cells in the spinal cord.

The NF‐κB pathway is essential for immune response regulation. The Tax protein from HTLV‐1 triggers this pathway, causing an increase in the production of pro‐inflammatory cytokines like TNF‐α, IL‐1β, and IL‐6. This leads to ongoing inflammation, which harms neurons and glial cells in the spinal cord. The constant activation of the NF‐κB pathway sustains the pathological immune response seen in HAM/TSP [109]. (Figure 1) However, it is still unclear how effective these inhibitors are in treating HAM/TSP patients clinically.

### 13.3 JAK/STAT Pathway

The JAK/STAT pathway is vital for cytokine signaling. In cells infected with HTLV‐1, there are changes in the expression of JAK/STAT pathway components, particularly STAT1. The activation of this pathway by the virus stimulates the transcription of genes linked to inflammation and immune responses. In HAM/TSP, the dysregulation of the JAK/STAT pathway results in the production of molecules that sustain an inflammatory environment, worsening the disease [110]. (Figure 1).

### 13.4 PI3K‐AKT Pathway

The PI3K‐AKT pathway is essential for controlling cell growth, survival, and metabolism. While it is less extensively studied in the context of HAM/TSP, evidence indicates that HTLV‐1 infection can activate this pathway. This activation may enhance the survival of infected cells and their resistance to programmed cell death (apoptosis), thereby maintaining the pool of infected cells and perpetuating chronic inflammation. Dysregulation of the PI3K‐AKT pathway can also affect the immune response and potentially play a role in the neurodegeneration observed in HAM/TSP [108]. (Figure 1)

Gene therapy is another promising approach to target HTLV‐1‐infected CD4 + T cells in HAM/TSP patients. Gene therapy involves the transfer of genetic material (DNA or RNA) to repair, regulate, or replace genes to cure a disease. Several studies have investigated gene therapy utilization in patients. One approach is to use zinc finger nucleases to specifically target and cleave the proviral DNA in infected cells. Its result was the reduction of the proviral load in vitro. However, the clinical efficacy of this approach remains to be determined [111].

Utilizing viral vectors for delivering therapeutic genes to infected cells is another approach to gene therapy. Various types of vectors, including adeno‐associated virus, adenovirus, and retrovirus‐based vectors could be utilized. However, these have not been very effective due to several limitations. These include inefficient transfer of genes, immune responses from the host against the viral vector, and the need for cells to be divided in order to facilitate transduction [112]. Lentiviral vectors pseudotyped with minimal filovirus envelopes have been shown to enhance gene transfer efficiency in murine lungs [112]. However, the safety and efficacy of these vectors in patients remain to be determined.

New developments in gene editing technology, like CRISPR‐Cas9, have shown promise in the field of gene therapy. CRISPR‐based gene therapy has been used to eliminate HIV from infected cells and to correct genetic mutations that cause inherited diseases [113]. Yet the effectiveness and safety of this therapy in HAM/TSP patients remain to be determined be ascertained. In another study, it has been showing that gene activation therapy using a recombinant retrovirus expressing HTLV‐1 Tax could induce apoptosis of infected cells and reduce viral load in a bovine model of HTLV‐1 infection [114].

Some studies have reported promising results of IFN‐ β1a for HAM/TSP. For instance, IFN‐1a therapy notably decreased the HTLV‐I tax mRNA load and reduced the presence of pathogenic tax‐specific CD8 cytotoxic T lymphocytes [115].

There are many gaps and uncertainties in translating these preclinical findings into clinical applications for patients. For example, there is a lack of standardized and validated methods for delivering and monitoring gene therapy in humans. There is also a risk of adverse effects or complications such as immune reactions, off‐target effects, insertional mutagenesis, or oncogenesis [116]. There is also a need for ethical approval and informed consent from patients and regulatory agencies. Therefore, more research and development are needed to establish the safety, effectiveness, and viability of gene therapy for patients.

The biosafety of gene therapy is an important consideration regarding the products. Several measures have been taken for improvement, including the development of safer viral vectors, and the use of non‐viral vectors and gene editing technologies that minimize off‐target effects [117].

Gene therapy is a method for addressing genetic disorders, with approved products like Zolgensma® for spinal muscular atrophy and liver‐targeted AAV8 vector‐mediated gene therapy for genetic liver disorders demonstrating success [111, 118]. These achievements have encouraged the exploration of gene therapy for other conditions, including HAM/TSP. One potential therapeutic target is CP‐690550, a JAK3 inhibitor that has shown inhibitory effects on cytokine‐mediated JAK3 activation and T‐cell proliferation in ex vivo studies involving patients. Additional research is required to assess the effectiveness of CP‐690550 in clinical applications for the treatment of HAM/TSP [105].

## 14 Protecting Neuronal Function

One of the main challenges in treating HAM/TSP is protecting neuronal function from the damaging effects of chronic inflammation and viral replication. Several neuroprotective agents have been investigated for their potential to improve motor function, bladder control, and life satisfaction in patients.

Nerve growth factor (NGF) is a neurotrophic protein that supports the survival, differentiation, and axonal regeneration of neurons [119]. NGF has been proposed as a neuroprotective agent for patients with severe symptoms, like muscle weakness, spasticity, and bladder dysfunction.

Erythropoietin (EPO) is a hormone that promotes red blood cell production, also known for its neuroprotective properties, which include mitigating inflammation, oxidative stress, and programmed cell death [120]. EPO has been used as a neuroprotective agent with moderate symptoms, such as gait disturbances and sensory deficits. In a small clinical trial, EPO was injected subcutaneously in patients, and enhancements in motor function and quality of life were observed in some patients [121, 122]. However, EPO injection was associated with hypertension, which required careful monitoring of blood pressure.

Pentosan polysulfate (PPS)‐ a semi‐synthetic polysaccharide‐ has anti‐inflammatory, anticoagulant, and neuroprotective effects, such as reducing oxidative stress, excitotoxicity, and demyelination [123]. PPS has been used as a neuroprotective agent for patients with mild symptoms, such as sensory disturbances and urinary dysfunction. In a small clinical trial, PPS was administered orally in patients, and improvements in motor function and bladder control were observed in some patients [124]. However, PPS administration was associated with diarrhea, which required dose adjustment and hydration. Both EPO and PPS are undefined that whether can have direct positive effect on HAM/TSP or not and needs further investigations.

Vitamin E is an antioxidant that scavenges ROS and inhibits lipid peroxidation, which plays a role in the development and progression of neurodegenerative disorders [125]. No significant side effects were reported with vitamin E administration.

Curcumin is a polyphenol that has anti‐inflammatory, antioxidant, and antiviral effects [126]. It has been used as an antioxidant agent for HAM/TSP patients with mild symptoms, such as joint pain and skin rash. In a small clinical trial, curcumin was administered orally in patients and enhancements in physical mobility and overall well‐being. Were observed in some patients [127, 128].

Tizanidine is an alpha‐2 adrenergic receptor agonist that inhibits excitatory neurotransmission and reduces spasticity, which is a common symptom and needs further research for effectiveness in these kinds of disorders including HAM/TSP [129].

## 15 Conclusion

HAM/TSP management needs a thorough and multidisciplinary approach that addresses the physical, social, psychological, and economic aspects of the disease. While antiretroviral therapy remains a good treatment option, several pharmacological approaches have been explored to target the immunological and inflammatory aspects of HAM/TSP. However, challenges exist in determining the optimal DMT and the limited effectiveness of current treatments, emphasizing the necessity for additional studies and well‐designed clinical trials.

Various pharmacological interventions have shown potential benefits in reducing inflammation, improving clinical symptoms, and decreasing proviral load in HAM/TSP patients. These include IFN‐alpha therapy, corticosteroids, and alternative medications such as prosultiamine, pentoxifylline, danazol, cyclosporine A, valproic acid, zidovudine and lamivudine, methylcobalamin, raltegravir, mirtazapine, famciclovir, cyclosporine, and myriadenolide. However, more research is needed to establish their efficacy, optimal dosages, and potential side effects. Also, Mogamulizumab is one of the most promising emerging treatment option.

Stem cell transplantation is also viable treatment alternatives for HAM/TSP. The administration of a blood transfusion can provide immediate relief of manifestations and improve the life quality, but it carries risks such as transfusion‐transmitted infections and adverse reactions. Stem cell therapy, especially HSCT, can be used to restore hematopoiesis and immunity in patients. However, the use of stem cell transplantation in HAM/TSP requires careful consideration of its risks and benefits.

Psychological and occupational therapies are vital components of the comprehensive management of HAM/TSP. Psychological support is crucial for addressing the mental health needs of patients who often experience depression, anxiety, and social isolation. Interventions such as cognitive‐behavioral therapy, supportive counseling, mindfulness‐based techniques, and group therapy can help patients cope with their symptoms and enhance their overall well‐being. Simultaneously, occupational therapy focuses on improving the physical functioning and independence of individuals with HAM/TSP. Through tailored exercise programs, assistive devices, and adaptive strategies, occupational therapy helps patients regain and maintain their abilities, promoting a better quality of life. By integrating these multidimensional approaches, healthcare providers can enhance the functionality, overall quality of life, and psychological well‐being, of patients.

## Author Contributions

**Meygol Mirzaei Rezaei:** writing – original draft, writing – review and editing. **Mahdi Khosravi Nia:** writing – original draft, investigation. **Kasra Allaei Rouzbahani:** writing – original draft, writing – review and editing. **Maryam Kazemi:** investigation, writing – original draft. **Negar Asghari Hosori:** writing – original draft. **Mehdi Norouzi:** Investigation, validation. **Farzaneh Sotoudegan:** investigation, validation. **Narges Eslami:** writing – review and editing. **Negar Ariamand:** writing – original draft, investigation. **Vahid Shahnavaz:** writing – original draft, investigation. **Arash Letafati:** writing – review and editing, validation, supervision, conceptualization. **Sayed‑Hamidreza Mozhgani:** supervision, validation, writing – review and editing, investigation.

## Ethics Statement

The authors have nothing to report.

## Consent

The authors have nothing to report.

## Conflicts of Interest

The authors declare no conflicts of interest.

## Transparency Statement

The Corresponding author affirms that this manuscript is an honest, accurate, and transparent account of the study being reported; that no important aspects of the study have been omitted; and that any discrepancies from the study as planned (and, if relevant, registered) have been explained.

## Data Availability

The data that support the findings of this study are available from the corresponding author upon reasonable request.
